# Role of ADAM10 and ADAM17 in Regulating CD137 Function

**DOI:** 10.3390/ijms22052730

**Published:** 2021-03-08

**Authors:** Jana Seidel, Sinje Leitzke, Björn Ahrens, Maria Sperrhacke, Sucharit Bhakdi, Karina Reiss

**Affiliations:** 1 Department of Dermatology, University of Kiel, 24105 Kiel, Germany; jseidel@dermatology.uni-kiel.de (J.S.); sleitzke@dermatology.uni-kiel.de (S.L.); bahrens@dermatology.uni-kiel.de (B.A.); msperrhacke@dermatology.uni-kiel.de (M.S.); 2 Independent Researcher, 24105 Kiel, Germany; sbhakdi@uni-mainz.de

**Keywords:** CD137, ADAM10, ADAM17, Anoctamin-6, T cell proliferation, cancer

## Abstract

Human CD137 (4-1BB), a member of the TNF receptor family, and its ligand CD137L (4-1BBL), are expressed on immune cells and tumor cells. CD137/CD137L interaction mediates bidirectional cellular responses of potential relevance in inflammatory diseases, autoimmunity and oncology. A soluble form of CD137 exists, elevated levels of which have been reported in patients with rheumatoid arthritis and various malignancies. Soluble CD137 (sCD137) is considered to represent a splice variant of CD137. In this report, however, evidence is presented that A Disintegrin and Metalloproteinase (ADAM)10 and potentially also ADAM17 are centrally involved in its generation. Release of sCD137 by transfected cell lines and primary T cells was uniformly inhibitable by ADAM10 inhibition. The shedding function of ADAM10 can be blocked through inhibition of its interaction with surface exposed phosphatidylserine (PS), and this effectively inhibited sCD137 generation. The phospholipid scramblase Anoctamin-6 (ANO6) traffics PS to the outer membrane and thus modifies ADAM10 function. Overexpression of ANO6 increased stimulated shedding, and hyperactive ANO6 led to maximal constitutive shedding of CD137. sCD137 was functionally active and augmented T cell proliferation. Our findings shed new light on the regulation of CD137/CD137L immune responses with potential impact on immunotherapeutic approaches targeting CD137.

## 1. Introduction

CD137 (also known as 4-1BB and TNFRSF9) is a type I transmembrane protein of the TNFR family that is widely expressed on cells of both the innate and adaptive immune system. It was originally discovered in screens for inducible genes in activated T cells [1,2]. Numerous studies devoted to its functional characterization on activated T cells and natural killer cells have been published implicating decisive potential of CD137 as a target for immunotherapies [3,4]. Interaction with its ligand CD137L results in upregulation of anti-apoptotic molecules, cytokine secretion, proliferation and enhanced effector function [5]. The type II transmembrane protein CD137L is constitutively expressed on several types of APCs such as mature dendritic cells and activated macrophages and transduces signals as well [6,7]. Rather remarkably, CD137L is also co-expressed with its receptor on activated T-lymphocytes [8,9,10].

Interaction of CD137 with its ligand on APCs promotes immune cell function against cancer [11,12]. Agonistic CD137 antibodies, e.g., urelumab and utomilumab, are being developed as immunotherapeutic agents to treat diverse malignancies [13].

Expression of CD137 and CD137L has also been reported for different types of cancer including lung cancer, leukemia and lymphoma [14,15,16]. The expression might be induced by activating K-ras mutations which are frequently observed, e.g., in pancreatic or colorectal cancer [17]. The biological significance of CD137 expression in cancer cells is presently unclear.

Recent reports have extended the possible involvement of the CD137/CD137L axis to a diversity of other pathologies including acute colitis [18] and atherosclerosis [19,20], and rheumatoid arthritis [21].

CD137 exists not only as a membrane-anchored protein but also as a soluble entity. Circulating CD137 has been detected in the serum of patients with colon cancer [22], leukemia and lymphoma [14]. Elevated soluble CD137 (sCD137) levels are indicative of an increased risk of cardiovascular events in patients with acute coronary syndrome [23,24] and correlate with disease severity in rheumatoid arthritis (RA) [21,25].

Like murine sCD137, human sCD137 is believed to be a splice variant. While one has been described in mouse, there are two in man [21,26,27]. However, many members of the TNFR family including TNFR1, TNFR2, OX40 (CD134), CD30, CD40, Death Receptor 4 (TRAILR1) and RANK are liberated and shed from cells through the action of metalloproteinases, predominant among which is ADAM17 [28,29,30,31,32,33].

Recently, we have presented evidence that transient exposure of the negatively charged phospholipid phosphatidylserine (PS) is pivotally involved in triggering sheddase function of ADAM17 as well as of ADAM10, its close homologue [34,35]. Surface-exposed PS interacts with cationic residues located in the stalk and membrane-proximal domains of ADAM10 and ADAM17, respectively. These electrostatic interactions are proposed to be critical for triggering sheddase function. PS resides predominantly in the inner leaflet of the plasma membrane in resting cells. Vertical traffic and translocation to the outer leaflet can be accelerated through the action of scramblases. Anoctamin-6 (ANO6), the best characterized calcium-activated scramblase, has been shown to play a key role in controlling the sheddase function of both ADAM10 and ADAM17 via its PS-translocating activity [36].

The functional effects of TNF-receptor shedding are multifaceted. Among others, the shedding event can serve to abrogate receptor-dependent signaling. Moreover, the soluble receptor might subserve decoy functions. Thus, it has been suggested that soluble TNFR1 can bind away and thus protect neighboring cells from the action of TNF-α [37,38,39,40].

Perhaps it is not just coincidence that ADAM10 and ADAM17 are also involved in the diseases mentioned above as well as in tumor development and progression [41,42,43].

Thus far, no study addressed the role of proteolysis as a regulatory event for CD137 function [27].

The aim of this study was to examine whether human CD137 (hCD137) could be released by proteolytic shedding. We show that this is indeed the case and identify ADAM10 as the major responsible proteinase, with ADAM17 assuming an additional potential role. In CD137- transfected cells, the shedding event could be stimulated by calcium influx and involved activation of phospholipid scramblase ANO6. CD137 endogenously expressed on activated primary human CD4+ and CD8+ T cells was also shed constitutively mainly through the action of ADAM10. Incubation of activated T cells with sCD137 led to enhanced T cell proliferation indicating that it can still bind its ligand. Shed CD137 may antagonize the effect of therapeutic CD137 agonists. Thus, our findings might be important for the development of new cancer immunotherapeutic strategies.

## 2. Results

### 2.1. ADAM10 Is the Major Sheddase of CD137 in HT29 Cells

**Figure 1:**
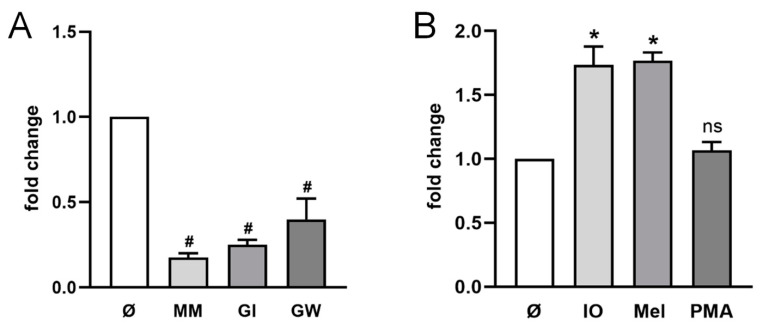
**Release of sCD137 is mediated by ADAM10 in HT29 cells.** (**A**,**B**) Cells were analyzed for the relative amount of shedding products in the supernatant in relation to total CD137 by ELISA shown as fold change compared to mock-treated cells. (**A**) Incubation with ADAM10 inhibitor GI (3 µM), ADAM10/17 inhibitor GW (3 µM) or broad-spectrum metalloprotease inhibitor marimastat (MM, 10 µM) for 24 h resulted in significantly reduced amounts of sCD137. (**B**) Cells were stimulated with ionomycin (IO; 1 µM), melittin (Mel; 1 µM) for 30 min or PMA (200 ng/mL) for 2 h. Stimulation with IO and Mel resulted in significantly increased CD137 shedding in HT29 cells. * indicates significant increase, # indicates significant decrease (*/# *p* < 0.05 (**A**) *n* = 4; (**B**) *n* = 3; ± s.e.m.) and ns = no significant difference compared to mock-treated cells (Ø). Data were analyzed by one-way analysis of variance and Bonferroni multiple comparison post hoc test.

The human colon adenocarcinoma cell line HT29 was employed for the first analysis of sCD137 release. The cells were transfected with CD137 plasmid and release of sCD137 was assessed in the presence of the broad spectrum-metalloprotease inhibitor marimastat (MM), the preferential ADAM10 inhibitor GI254023X (GI), or GW280264 (GW), an inhibitor of both ADAM10 and ADAM17 (Figure 1A). All three inhibitors led to a significant reduction in sCD137 in the supernatant. This was the first indication that metalloproteinases could cleave and release CD137 from cells, and that ADAM10 was the major sheddase involved.

ADAM10- and ADAM17-mediated shedding can be differentially activated dependent on stimuli. ADAM10 activity can be triggered by induction of calcium-influx, the membrane-active cationic peptide melittin can upregulate both ADAM10 and ADAM17 function, while the phorbol ester PMA is a classical selective stimulus of ADAM17 [44,45,46]. As shown in Figure 1B, enhanced shedding of CD137 was induced by ionomycin and melittin but not by PMA, indicating that ADAM10 represents the major sheddase that is also responsible for stimulated shedding.

CD137 transfection was controlled in parallel by immunoblot (Supplementary Figure S1A). Total amounts of CD137 can be found in Supplementary Figure S1B,C.

### 2.2. ADAM10 and ADAM17 Can Release CD137 in HEK Cells

**Figure 2:**
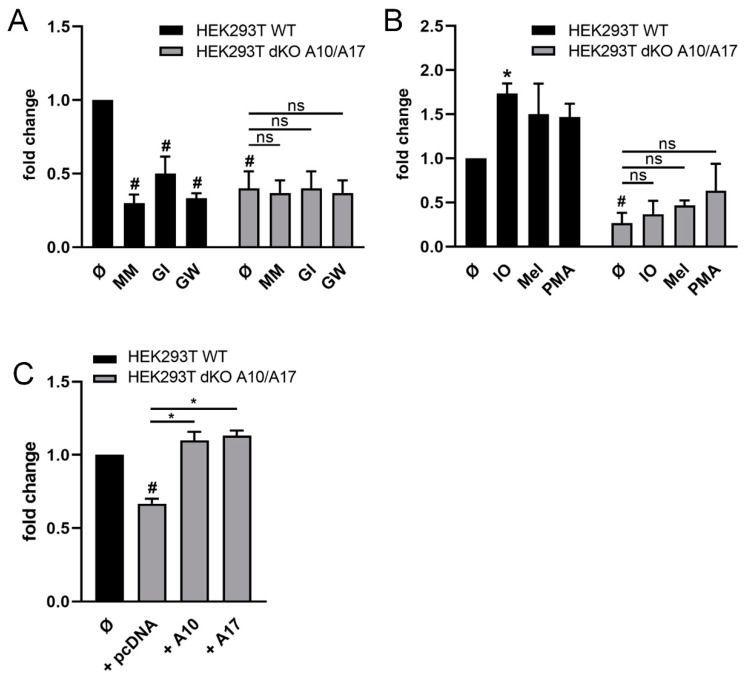
**ADAM10 and ADAM17 mediate CD137 release in HEK cells.** (**A**,**B**) Cells were analyzed for the relative amount of shedding products in the supernatant in relation to total CD137 by ELISA shown as fold change compared to mock-treated cells. (**A**) Release of CD137 was significantly decreased in mock-treated HEK293T dKO A10/17 cells compared to WT cells. Incubation with metalloprotease inhibitors GI (3 µM), GW (3 µM) and MM (10 µM) for 24 h resulted in a significantly reduced amount of sCD137 in WT cells but not in HEK293T A10/A17 dKO cells. (**B**) Cells were stimulated with ionomycin (IO, 1 µM), melittin (Mel; 1 µM) for 30 min or PMA (200 ng/mL) for 2 h. Stimulation with ionomycin resulted in significantly increased shedding of CD137 in HEK293T WT cells. No significant difference was observed in HEK293T dKO A10/A17 cells. (**C**) HEK293T dKO A10/A17 cells were co-transfected with CD137 and ADAM10, ADAM17 or mock vector and analyzed by CD137 ELISA. Re-Transfection of ADAM10 and ADAM17 resulted in a significant increase in CD137 shedding in dKO A10/A17 cells. * indicates significant increase, # indicates significant decrease compared to mock-transfected cells (Ø) (*/# *p* < 0.05 (**A**–**C**) *n* = 3; ± s.e.m.). ns = no significant difference. Data were analyzed by one-way (**C**) or two-way (**A**,**B**) analysis of variance and Bonferroni multiple comparison post hoc test.

Studies addressing the individual roles of ADAM10/ADAM17 are aided by the availability of double-deficient (dKO) HEK293T cells [47]. Wild-type (WT) and dKO-cells were transfected with CD137 and shedding was analyzed in the presence of the different inhibitors. As shown in Figure 2A, release of sCD137 occurred constitutively in WT-cells, and shedding was significantly reduced in the presence of MM, the ADAM17/ADAM10 inhibitor GW, and the ADAM10 inhibitor GI. Constitutive CD137 release was conspicuously lower in double-deficient HEK cells compared with WT-cells and was not further decreased in the presence of inhibitors.

Stimulation experiments provided data that complemented and corroborated the findings. As shown in Figure 2B, shedding in WT-cells was significantly increased upon ionomycin (IO) stimulation and also enhanced upon exposure to melittin, whereas no responses were noted in the double-deficient cells. Finally, it was found that re-transfection of double knock-out cells with ADAM10 restored shedding capacity (Figure 2C). Retransfection with ADAM17 had a similar effect (Figure 2C). This was in line with earlier reports that, in the absence of one protease, ADAM10 and ADAM17 can replace each other in cleaving substrates such as TGF-.

CD137 transfection was controlled in parallel by immunoblot (Supplementary Figure S2A). Total amounts of CD137 can be found in Supplementary Figure S2B–D.

### 2.3. Ionomycin-Induced Shedding of CD137 Is PS Dependent

Recently, we have presented evidence that the interaction of ADAM10 and ADAM17 with externalized phosphatidylserine (PS) is required for the proteases to exert their sheddase function [34,35,48]. To discern whether stimulated shedding of CD137 was also PS-dependent, competition experiments were conducted employing phosphorylserine (OPS, O-phospho-l-serine), the soluble headgroup of PS, or lactadherin (LA), which binds to surface-exposed PS [49,50] and thus blocks its accessibility to other PS-binders.

**Figure 3:**
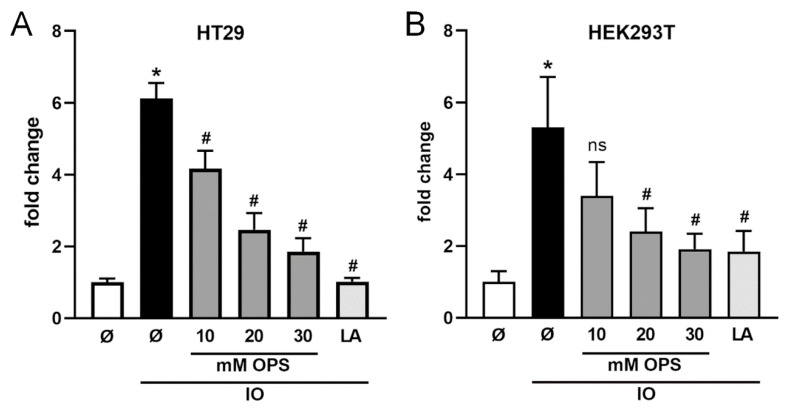
**Ionomycin-induced shedding of CD137 depends on ADAM-phosphatidylserine (PS) interaction.** CD137-transfected HT29 cells (**A**) or HEK293T WT cells (**B**) were stimulated with ionomycin (IO; 1 µM) for 30 min in the presence of OPS or lactadherin (LA, 2 µM) and analyzed for the relative amount of shedding products in the supernatant by ELISA. OPS and lactadherin significantly reduced CD137-shedding. * indicates significant increase compared to mock-treated cells, # indicates significant decrease (*/# *p* < 0.05 (**A**) *n* = 4; (**B**) *n* = 5; ± s.e.m.) or ns = no significant difference compared to IO-stimulated cells. Data were analyzed by one-way analysis of variance and Holm-Sidak multiple comparison post hoc test.

HT29 and HEK cells were stimulated in the absence or presence of the inhibitors and released sCD137 was quantified. OPS dose-dependently reduced levels of sCD137 in the supernatants of both cell lines (Figure 3A,B). In corroboration of this finding, lactadherin used at the entirely non-toxic concentration of 2 µM exerted maximal inhibition [35,36]. Total amounts of CD137 can be found in Supplementary Figure S3A,B.

### 2.4. Anoctamin-6 Modulates CD137 Release

ANO6 activation through calcium-elevation results in rapid translocation of PS to the external membrane leaflet, which triggers ADAM10/17 sheddase function. Transfection experiments were next undertaken with ANO6 and with a point substitution mutant D408G that is uniquely hypersensitive to calcium [36]. This hypersensitivity leads to unrestrained activity and, in consequence, to permanent PS-exposure and continued ADAM10 activation in resting cells [36].

**Figure 4:**
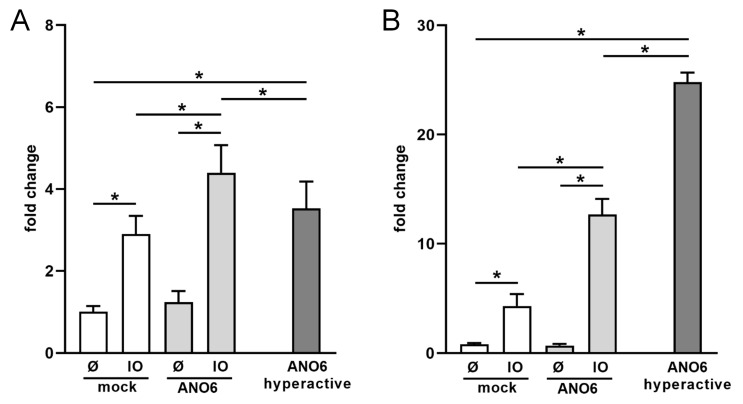
**Anoctamin-6 (ANO6) modulates CD137 shedding. ANO6 and mock-transfected** (**A**) HT29 cells or (**B**) HEK293T WT cells were stimulated with ionomycin (IO; 1 µM) for 30 min. In addition, cells co-transfected with CD137 and hyperactive ANO6 were analyzed in the absence of any stimulus for 30 min. The relative amount of shedding products in the supernatant was determined in relation to total CD137 by ELISA. IO-induced shedding was significantly increased upon overexpression of ANO6. Shedding of CD137 was significantly increased upon overexpression of hyperactive ANO6 compared to mock-transfected non-stimulated cells. * indicates significant increase (* *p* < 0.05 (**A**) *n* = 5; (**B**) *n* = 3; ± s.e.m.). Data were analyzed by one-way analysis of variance and Holm–Sidak multiple comparison post hoc test.

Figure 4A depicts the results of experiments with HT29 cells. Transfection with ANO6 did not primarily affect levels of constitutively released sCD137. However, the release was significantly increased relative to controls upon IO-stimulation. Very conspicuously, cells transfected with the hyperactive ANO6-mutant constitutively shed large amounts of CD137 in the absence of any stimulus.

Figure 4B shows the data obtained with HEK cells. The findings followed the same pattern but were yet more pronounced. Stimulated shedding in ANO6-transfected cells was augmented threefold, and transfection of the hyperactive mutant led to constitute release of sCD137 to even higher levels. Total amounts of CD137 can be found in Supplementary Figure S4.

### 2.5. Release of Endogenously Expressed CD137 in T Cells

**Figure 5:**
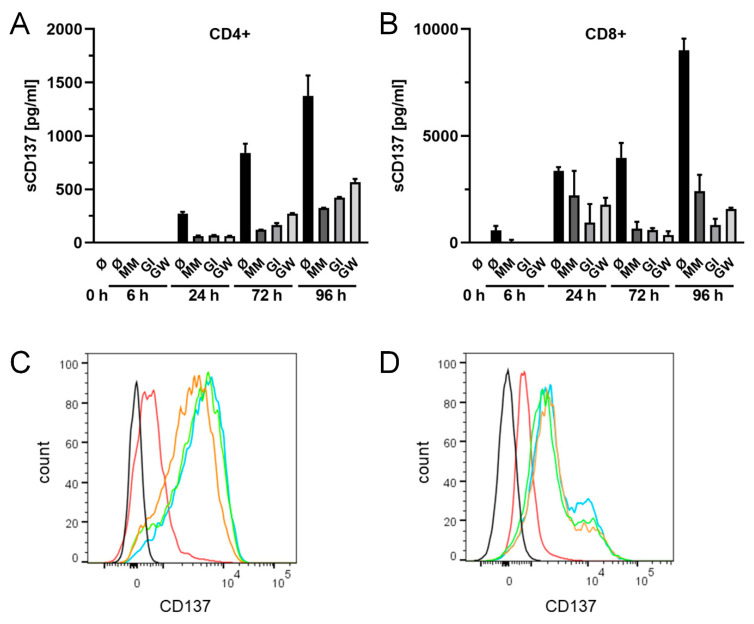
**ADAM10 is the major sheddase of endogenously expressed CD137 in T cells.** (**A/C**) Primary CD4^+^ T cells or (**B/D**) CD8^+^ T cells were treated with ADAM10 inhibitor GI (3 µM), mixed ADAM10/ADAM17 inhibitor GW (3 µM) or broad-spectrum metalloprotease inhibitor marimastat (MM, 10 µM) for 6, 24, 72 or 96 h. (**A/B**) Supernatants were analyzed for soluble shedding products of CD137 with ELISA. Treatment with GI, GW and MM resulted in a strikingly reduced amount of sCD137. One representative experiment out of five experiments is shown with mean ± SD. (**C/D**) Expression of cell surface CD137 on CD4^+^ and CD8^+^ T cells was analyzed using FITC labeled anti-CD137 mAb 72 h after activation. Incubation with MM (blue), GW (orange) and GI (green) increased the amount of cell bound CD137 compared to untreated cells (red). Black line: mock-stained control. One representative out of three experiments is shown.

Expression of CD137 is best characterized in T cells, where it can readily be induced by their activation [1,51]. CD4^+^ and CD8^+^ T cells were activated with soluble CD3- and CD28-antibodies. Expression of CD137 was detected 6 h in CD8+ cells and 24 h in CD4^+^ cells following stimulation. Soluble CD137 was found to accumulate over time. Addition of the metalloprotease inhibitors marimastat, GI or GW to the media strikingly reduced levels of sCD137 in supernatants of both cell types (Figure 5A,B). The reduction in sCD137 was accompanied by an increase in CD137 on the surface of the activated CD4^+^ and CD8^+^ cells (Figure 5C,D). These findings show that ADAM10 is the principle effector of sCD137 release from human lymphocytes.

### 2.6. Shed sCD137 Induces Proliferation of Activated T Cells

Cleavage of ADAM10 substrates always occurs close to the membrane in the stalk region of the respective substrate. Therefore, it can be assumed that shed sCD137 encompasses the extracellular domain and might retain the ability to bind to CD137 ligand.

**Figure 6:**
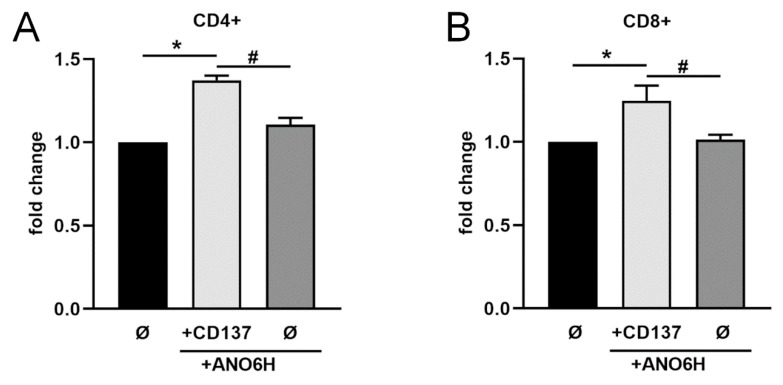
**Shed sCD137 augments proliferation of CD4+ and CD8+ T cells**. Proliferation assay of primary (**A**) CD4^+^ T cells or (**B**) CD8^+^ T cells. WT HEK293T cells were either co-transfected with CD137 and mock vector, CD137 and hyperactive ANO6 (ANO6H), or ANO6H and mock vector. Then, 24 h after transfection, medium was changed and supernatants were harvested after further 24 h. Pre-activated T cells were cultured in the presence of the precleared conditioned HEK cell medium for 72 h and proliferation was analyzed. Untreated control cells were standardized to baseline. * indicates significant increase compared to mock-treated cells, # indicates significant decrease (*/# *p* < 0.05 (**A**,**B**) *n* = 4; ± s.e.m.). Data were analyzed by one-way analysis of variance and Sidak multiple comparison post hoc test.

Interestingly, T cells do not only express CD137 but also CD137L upon activation [8,9,10], (see also Supplementary Figure S5). Activation of CD137L through receptor binding might lead to pro- and anti-inflammatory effects as well [5]. Thus, we addressed the question how shed CD137 would affect T cell proliferation. According to our previous findings, huge amounts of sCD137 were released from transfected HEK cells in the presence of hyperactive ANO6 without any stimulus. Thus, we made use of the conditioned medium of CD137/hyperactive ANO6 co-transfected cells and mock-transfected cells or cells transfected with hyperactive ANO6 alone. When T cells were incubated in medium containing sCD137, T cell proliferation was significantly augmented (Figure 6). These data indicate that shed sCD137 is functionally active and able to bind to CD137L expressing cells.

## 3. Discussion

The TNFR-superfamily assumes a central position in the signaling network and functioning of the immune system. Many studies have been undertaken to unravel the role of the CD137/CD137L axis and its links to pathophysiological immune processes. An emerging topic relates to the function and relevance of membrane-anchored CD137 and its soluble counterpart sCD137. Elevated plasma levels of the latter have been found in patients with various malignancies as well as in patients with rheumatoid arthritis, where levels have been found to be associated with disease severity.

Current evidence indicates that sCD137 is a spliced variant of the receptor, whose expression is subject to regulation at the transcriptional level. Nevertheless, we deemed it worthwhile to investigate whether an alternative mechanism for sCD137 generation might exist that would allow for rapid, pliable modulation of its release from cells. From the beginning, similarities were noted between findings reported for sCD137 and soluble CD27 (sCD27), another member of the TNFR-superfamily. High levels of sCD27 have also been found in patients suffering from rheumatoid arthritis [52]. In vivo levels of sCD27 correlate with tumor load in patients with leukemia and lymphoma [52,53]. Huang et al. reported that sCD27 increased T cell activation and proliferation in vitro and in vivo [54]. The authors speculate that this activation might modulate tumor immunity.

sCD27 is known to be released by metalloproteinase-mediated cleavage of the membrane-anchored molecule, so the suspicion arose that the same might hold for sCD137. Application of metalloproteinase inhibitor has been reported to increase amounts of cell-bound CD137 in murine lymphocytes [31], a finding that constitutes evidence for the notion of proteolytic shedding.

The collective results reported herein now leave little room for doubt that sCD137 is indeed generated mainly through proteolytic release by ADAM10. In the first set of experiments, constitutive and stimulated shedding was analyzed in CD137-transfected HEK and HT29 cells. In both cell lines, constitutive release of sCD137 was significantly diminished in the presence of a broad-spectrum metalloprotease inhibitor. Incubation with the preferential ADAM10 inhibitor GI led to a comparable reduction which indicated that ADAM10 is the major metalloprotease involved in CD137 shedding. ADAM10-sheddase function is enhanced through induction of calcium-influx, which can be provoked by treatment of cells with calcium-ionophore and melittin. Both agents strongly augmented CD137 shedding. Employment of HEK cells deficient in ADAM10 and ADAM17 rounded up the findings. Constitutive release of sCD137 fell to low levels and neither ionomycin nor melittin stimulated shedding capacity, which was restored upon re-transfection of the cells with ADAM10. Interestingly, some substrates are cleaved by ADAM10 as well as ADAM17 depending on the cell type and stimulus. While ADAM17 is the major sheddase of TGF-α under normal conditions, ADAM10 releases this substrate when ADAM17 is absent [46]. Both, ADAM10 and ADAM17 are also involved in the shedding of the T cell immunoglobulin and mucin domain 3 (TIM3) protein from activated T cells [55]. Tim-3 is shed by ADAM10 and ADAM17 after PMA or ionomycin stimulation, respectively. In monocytes, ADAM10 and to a lesser extent ADAM17 were found to be responsible for LPS-induced down-regulation of Tim-3. In our study, re-transfection of ADAM17 also restored the release of CD137 in ADAM10/ADAM17 dKO cells. Thus, both proteases are in principle able to shed this protein.

Calcium-influx triggers scramblase activation resulting in exposure of PS at the membrane surface. According to the 3D structure of the extracellular domain [56] the ADAM10 active site is occluded by a short peptide loop located at the commencement of the stalk region. Attraction of the cationic PS-binding motif to surface exposed PS may thus serve to draw this loop out of the catalytic site enabling substrate access. This event is suppressed in the presence of the soluble phospholipid head group or through blockade of surface-exposed PS with lactadherin. Both, in HT29 and HEK cells, ionomycin-provoked enhancement of substrate cleavage was dose-dependently reduced by OPS and virtually abrogated by lactadherin.

Experiments followed in which cells were transfected with ANO6 to enhance expression of the major calcium-activated scramblase that translocates PS molecules from the inner to the outer membrane leaflet. Transfection was also undertaken with the hyperactive D408G substitution mutant. Overexpression of WT ANO6 did not alter constitutional shedding of CD137, but upon stimulation with calcium ionophore, however, ANO6-transfected cells shed significantly higher amounts of sCD137 into the supernatants. The behavior of cells transfected with the ANO6 hyperactive mutant was remarkable. Maximal shedding of sCD137 occurred constitutively in both HT29 and HEK cells in the absence of any stimulus. Transfection of CD137 encoding plasmid in the HT29 and HEK cell lines ruled out the contribution of alternative splicing in the generation of sCD137.

Currently, the major fraction of soluble CD137 is believed to represent the secreted splice form that is expressed by activated T cells [26]. Thus, a central question arose whether sCD137 release by these cells also involved ADAM10 or ADAM17. CD4+ and CD8+ T cells were activated and cultured in the presence or absence of ADAM inhibitors. sCD137 accumulated to high levels during the observation period of 96 h, particularly in supernatants of CD8+ lymphocytes, and release was effectively reduced by all inhibitors. These findings leave little room for doubt that generation of sCD137 in human lymphocytes also occurs mainly through the action of ADAM10. Additional experiments addressing the contribution of the spliced form in comparison to ADAM-derived soluble CD137 would be of high interest.

Rapid, regulated shedding of CD137 must therefore now be considered in the context of the function of the CD137/sCD137 axis with its multiple facets [5]. The soluble spliced form of CD137 is known to bind to its ligand. Studies are currently underway to examine the consequences, which can be expected to be complex. Interaction of cell-bound CD137 with CD137L elicits bidirectional signaling in participating cells. Tu et al. have reported that soluble CD137 also provokes reversed signaling. sCD137 was found to represent a chemotactic factor for monocytes and Jurkat T-cells that express the ligand CD137L [57].

An analogous finding was made in this study. Co-transfection of HEK cells with CD137 and hyperactive ANO6 led to vigorous constitutional shedding of sCD137 into cell supernatants. The conditioned media were found to provide a stimulus for activated lymphocytes that are known to express CD137L. The effect was not noted when HEK cells were transfected with hyperactive ANO6 alone.

This result was somewhat unexpected in the light of previous data of Eun et al. [58], who reported that CD137 ligand signaling to T cells limited T cell activation. However, their observation pertained only to murine T-cells activated with very low concentration of 0.1 µg/mL anti-CD3. The effect was much less apparent at higher concentrations of antibody. It will be of interest to analyze and compare the human and murine CD137/CD137L axis in the future. This will be important for interpretation of in vivo data obtained in animal models.

Cellular ATP-content was employed as the readout for activation as it is routinely used to assess T cell numbers [59,60,61]. ATP-increase may also derive from enhanced mitochondrial function. Interestingly, this has been described as a costimulatory effect of CD137 activation in T cells [62,63].

It is clear that the functional data presented herein are no more than preliminary and must be followed by in-depth analyses of underlying signaling pathways and cell biological events. The prime intent of this report is to draw general attention to the existing link between two major signaling networks of immune cells and malignant cells. Indeed, tumor biology will possibly become a particularly rewarding field for future research. Sophisticated ruses are employed by tumor cells for immune evasion. One is the upregulation and cell surface expression of molecules such as PD-L1 (programmed death ligand 1) and CTLA-4 (cytotoxic T-lymphocyte associated protein 4) whose interaction with the respective partners on immune cells have detrimental consequences.

Possibly, CD137/CD137L will eventually join the list of such molecules. CD137 is being detected on an ever-increasing number of tumors. It may be more than coincidence that overexpression of ADAM10 contributes to tumor development [43], and that hypoxic conditions induce increased expression of both the secreted CD137 spliced variant [64] and ADAM10. It is tempting to speculate, for example, that soluble CD137 functions as a decoy and competitor of CD137 to negatively impact on physiological functions of the CD137/CD137L axis. With the present identification of ADAM10 as the major generator of soluble CD137, a new link is provided that should prove useful to further research in the field.

## 4. Materials and Methods

### 4.1. Materials

O-phospho-L-serine (OPS) and Phorbol 12-myristate-13-acetate (PMA) were obtained from Sigma Aldrich (St. Louis, MO, USA). Ionomycin was purchased from Merck Millipore (Darmstadt, Germany). Lactadherin was from Haematologic Technologies (Essex Junction, Vermont, USA). Hydroxamate-based ADAM17/ADAM10 inhibitor GW280264X [65] was purchased from Aeobious (Gloucester, MA, USA). Marimastat and ADAM10 inhibitor GI254023X [66] were purchased from Tocris Bioscience (Bristol, UK). Melittin was synthesized as described [67]. Anti-turboGFP antibody was from Invitrogen (PA5-22688, Carlsbad, CA, USA), anti-tubulin clone E7 was from DSHB, Iowa City, IA, USA. Secondary antibodies for automated Western were obtained from Protein Simple (San Jose, CA, USA).

### 4.2. Cell Culture

HEK293T and HT29 cells were purchased from Sigma Aldrich (St. Louis, MO, USA). ADAM10/ADAM17 double-deficient HEK293T cells (dKO) have been described before [47]. HEK293T, HT29 and ADAM10/ADAM17 double-deficient HEK293T cells were grown in high glucose DMEM, Thermo Fisher Scientific (Waltham, MA, USA) supplemented with 10% fetal calf serum (FCS), PAA Laboratories (Cölde, Germany) and 1% penicillin/streptomycin (Pen/Strep), PAA Laboratories (Cölde, Germany).

### 4.3. T Cell Isolation and FACS Analysis

Peripheral blood mononuclear cells (PBMC) were isolated from leukocyte concentrates of healthy adult blood donors obtained from the Institute of Transfusion Medicine, University Hospital Schleswig-Holstein (Kiel, Germany) by Ficoll density gradient centrifugation, Sigma Aldrich (St. Louis, MO, USA).

CD4^+^ or CD8^+^ T Cells were isolated from PBMCs according to manufacturer’s recommendations using CD4^+^ or CD8^+^ microbeads, Miltenyi Biotec (Bergisch Gladbach, Germany) or Nanobeads, Biolegend (San Diego, CA, USA). Cells were grown in RPMI-1640 medium, Thermo Fisher Scientific (Waltham, MA, USA) supplemented with 10% FCS and 1% Pen/Strep and activated with anti CD3- and CD28 antibodies (1 µg/mL; Miltenyi Biotec, Bergisch Gladbach, Germany).

Cells were stained in different combinations with the following antibodies according to manufacturer’s recommendations: CD4-APC-Vio-770 (M-T466), CD8-VioBlue (REA734) and CD137-VioBright-FITC (REA765), all Miltenyi Biotech (Bergisch Gladbach, Germany). Propidium iodide, Novus Biological (Centennial, CO, USA), was used to exclude dead cells. Staining was done according to manufacturer’s protocol. All data were acquired on a Cytoflex, Beckmann Coulter (Brea, CA, USA). FlowJo, TreeStar (Ashland, OR, USA), was used for data analysis.

### 4.4. Expression Vectors and Transfection

The expression vector for human CD137-GFP was purchased from OriGene, (Rockville, MD, USA. The expression vector for murine ADAM17 was from Gillian Murphy (Cambridge, UK). The expression vector for murine ADAM10 was described before. ANO6-WT plasmid (EX-I1781-M02) was purchased from GeneCopoeia, (Rockville, MD, USA. The hyperactive D408G ANO6 mutant was generated by point mutation of WT ANO6 as described in Veit et al. [36].

HEK293T, HT29 and ADAM10/ADAM17 double-deficient HEK293T cells were transfected using Turbofect Transfection Reagent, Thermo Fisher Scientific (Waltham, MA, USA), according to the manufacturer’s instructions. Then, 24 h after transfection of expression vectors, cell medium was replaced by fresh DMEM. Transfection efficiency of CD137 was always controlled in parallel by Simple Western analyses (Supplementary Figures S1 and S2).

### 4.5. Enzyme-Linked Immunosorbent Assay

CD137 ELISA (R&D, Minneapolis, MN, USA) was performed according to the manufacturer’s instructions. HEK293T or HT29 cells were grown until confluence in 6- or 12-well plates. For ADAM10 and/or ADAM17 inhibition experiments cells were treated with marimastat (10 µM) GI254023X (3 µM) or GW280264X (3 µM) over 24 h. For stimulation assays, cells were incubated with ionomycin (1 µM), melittin (1 µM) for 30 min or PMA (200 ng/mL) for 2 h. Preincubation with OPS (10 mM, 20 mM, 30 mM) or Lactadherin (2 µM) was done for 15 min and followed by stimulation with ionomycin (1 µM) for 30 min. CD137 ELISA was performed with supernatants and cell lysates. Cells were lysed in lysis buffer (5 mM Tris-HCl (pH 7.5), 1 mM EGTA, 250 mM saccharose, 1% Triton X-100) supplemented with cOmplete inhibitor cocktail, Roche Applied Science (Penzberg, Germany), and 10 mM 1,10-phenanthroline monohydrate [68]. Supernatants and lysates were analyzed in duplicates. The relative amount of shedding products in the supernatant was calculated in relation to total CD137 (supernatant and lysate).

### 4.6. Proliferation Assay

To investigate the effect of shed CD137 on T cell proliferation CD4^+^ and CD8^+^ T cells were incubated with conditioned medium of transfected HEK293T cells. The latter were transfected with mock vectors, hyperactive ANO6 or with CD137 and hyperactive ANO6. Then, 24 h after transfection, medium was exchanged. Supernatants were collected after 24 h and precleared of cells, debris and larger vesicles by centrifugation. Primary T cells were pre-activated with anti-CD3/CD18 antibodies for 24 h and seeded into 96 well plates in 50 µL medium. Then, conditioned medium of HEK cells was added 1:1. After 72 h the cell number was determined using Cell Titer Glo Luminescent Cell Viability Assay, Promega (Fitchburg WI, USA), according to the manufacturer’s instructions.

### 4.7. Semi-Quantitative RT-PCR

The expression of CD137 and CD137L mRNA in CD4^+^ and CD8^+^ T-cells was detected using RT-PCR. THP1 cells were used as control. RNA was isolated using NucleoSpin RNA Kit (Macherey-Nagel, Düren, Germany) following the manufacturer’s instructions. RNA (1 µg) was reverse-transcribed using the PrimeScript™ RT Master Mix Kit (TaKaRa, Kusatsu, Shiga, Japan). The following primers were used to amplify human CD137 cDNA: sense primer, 5’-TTGCTGGTCCTCAACTTTGA-3’; and antisense primer, 5’-AACACCTTTACACTGCCTGC-3’ and for human CD137L cDNA: sense primer, 5’-GCCCAAAATGTTCTGCTGAT-3’ and antisense primer, 5’-CAGCTCCTTCGTGTCCTCTT-3’. The housekeeping gene HPRT1 served as a control: sense primer, 5’-TGGCGTCGTGATTAGTGATG-3’; antisense primer, 5’-TCTCGAGCAAGACGTTCAGT-3’. All primers were purchased from Sigma Aldrich, St. Louis, Missouri, USA. After denaturation at 95 °C for 30 s, 6 cycles of PCR were performed, each consisting of a denaturation step (95 °C for 20 s), an annealing step (68–1 °C/cycle for 30 s), and an elongation step (68 °C for 20 s), followed by 28  cycles, each consisting of a denaturation step (95 °C for 20 s), an annealing step (62 °C for 30 s), and an elongation step (68 °C for 30 s), followed by a final extension at 68 °C for 5 min. The PCR products of CD137, CD137L and HPRT1 were separated by electrophoresis on a 2.5% agarose gel containing GelRed (Biotium, Fremont, CA, USA) and then photographed under ultraviolet light.

### 4.8. Automated Western

Cells were lysed in lysis buffer (5 mMTris-HCl (pH 7.5), 1 mM EGTA, 250 mM saccharose, 1% Triton X-100) supplemented with complete inhibitor cocktail (Roche Applied Science, Penzberg, Germany) and 10 mM 1,10-phenanthroline monohydrate. Automated Western was performed with equal concentrations of protein per sample using WES™ (Protein Simple, San Jose, CA, USA) according to the manufacturer’s instructions. Detection of CD137 in HT29 and HEK cells via Simple Western is shown in Supplementary Figures S1 and S2 using anti-tGFP antibodies.

### 4.9. Statistical Analysis

All values for the ectodomain shedding assays are expressed as means ± standard error of the mean. The standard error values indicate the variation between mean values obtained from at least three independent experiments. Statistics were generated using one-way or two-way analysis of variance (one-way/two-way ANOVA) and multiple comparison post hoc test as designated. *p* values < 0.05 were considered statistically significant (either indicated with * or #).

## Supplementary Materials

The following are available online at https://www.mdpi.com/1422-0067/22/5/2730/s1.
