# Supplementary material for: Role of ADAM10 and ADAM17 in Regulating CD137 Function
Source: Int J Mol Sci. 2021 Mar 8;22(5):2730. doi: 10.3390/ijms22052730 (PMC7962946; doi:10.3390/ijms22052730)
Supplement: Supplementary file 1 [file ijms-22-02730-s001.pdf]

Supplementary Figure S1

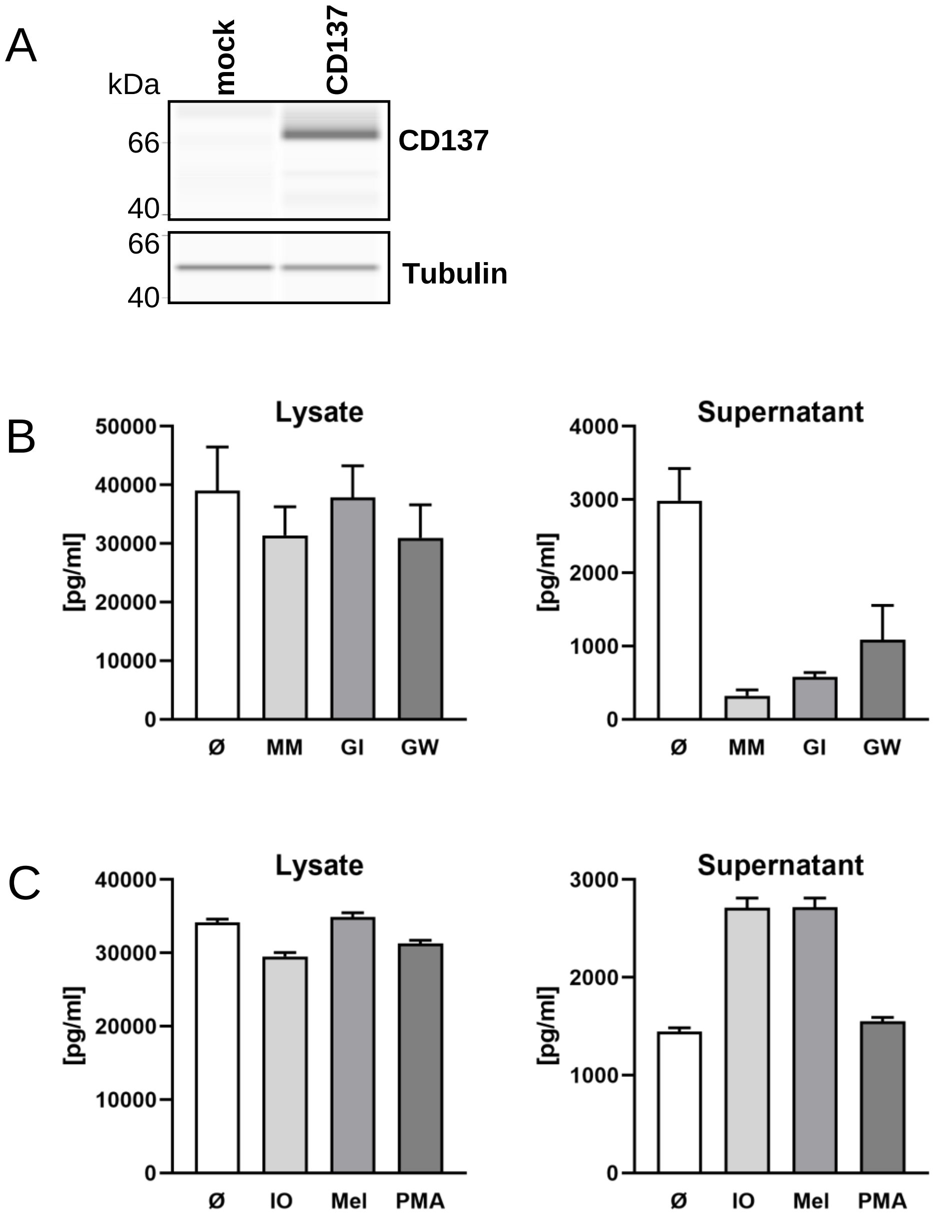

Supplementary Figure S1: Release of sCD137 is mediated by ADAM10 in HT29 cells. (A) Transfection of CD137 was controlled by Simple Western using anti-tGFP antibodies. Tubulin was used as loading control. **(B, C)** One representative experiment out of three ELISAs (shown normalized in Figure 1A and B) is depicted to show the absolute levels of CD137 in cell lysates and supernatants, respectively (mean +/- SD). **(B)** Incubation with ADAM10 inhibitor GI (3 µM), ADAM10/17 inhibitor GW (3 µM) or broad-spectrum metalloprotease inhibitor marimastat (MM, 10 µM) resulted in reduced amounts of sCD137 in the supernatants. **(B)** Cells were stimulated with ionomycin (IO; 1 µM), melittin (Mel; 1 µM) for 30 min or with PMA (200 ng/ml) for 2 h. Cell lysates and supernatants were analyzed by CD137 ELISA.

Supplementary Figure S2

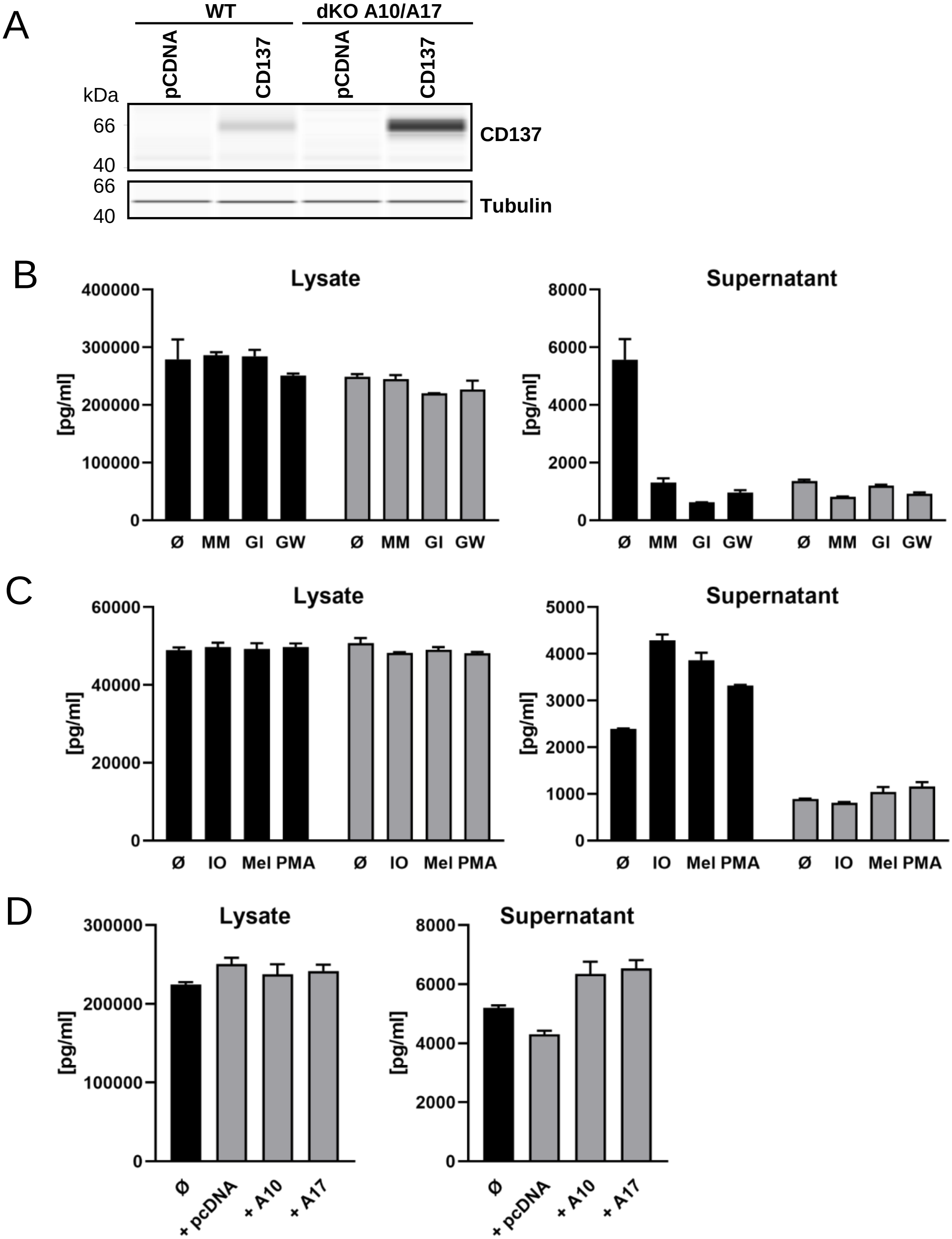

Supplementary Figure S2: ADAM10 and ADAM17 mediate CD137 release in HEK cells. (A) Transfection of CD137 in wild-type (WT) HEK and ADAM17/ADAM17 double-knockout HEK cells (dKO) was controlled by Simple Western using anti-tGFP antibodies. Tubulin was used as loading control. (B-D). One representative experiment out of three ELISAs (shown normalized in Figure 2A-C) is depicted to show the absolute levels of CD137 in cell lysates and supernatants of WT (black bars) and dKO cells (grey bars), respectively (mean +/- SD). **(B)** Incubation with ADAM10 inhibitor GI (3 µM), ADAM10/17 inhibitor GW (3 µM) or broad-spectrum metalloprotease inhibitor marimastat (MM, 10 µM) resulted in reduced amounts of sCD137 in the supernatants of WT cells. **(C)** Cells were stimulated with ionomycin (IO; 1 µM) or melittin (Mel; 1 µM) for 30 min or with PMA (200 ng/ml) for 2 h. **(D)** HEK293T dKO A10/A17 cells were co-transfected with CD137 and ADAM10, ADAM17 or mock vector and analyzed by CD137 ELISA.

Supplementary Figure S3

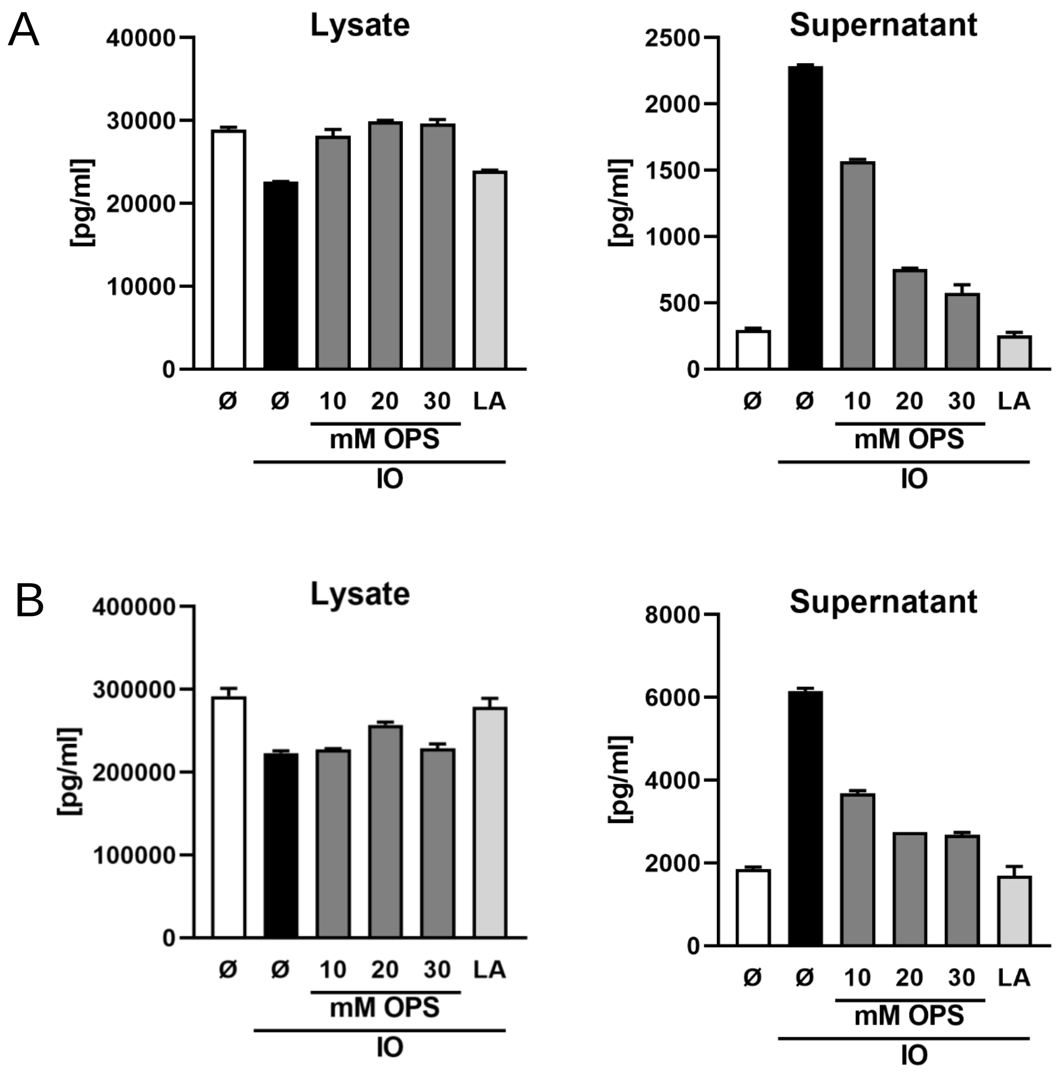

Supplementary Figure S3: Ionomycin-induced sheding of CD137 depends on ADAM-phosphatidylserine (PS) interaction. One representative experiment (shown normalized in Figure 3A/B) is depicted to show the absolute levels of CD137 in cell lysates and supernatants of **(A)** HT29 (n=4) and **(B)** HEK cells (n=5), respectively. CD137-transfected HT29 cells or HEK293T WT cells were stimulated with ionomycin (IO; 1 µM) for 30 min in the presence of OPS or lactadherin (LA, 2 µM). Cells and supernatants were analyzed by CD137 ELISA (mean +/- SD).

Supplementary Figure S4

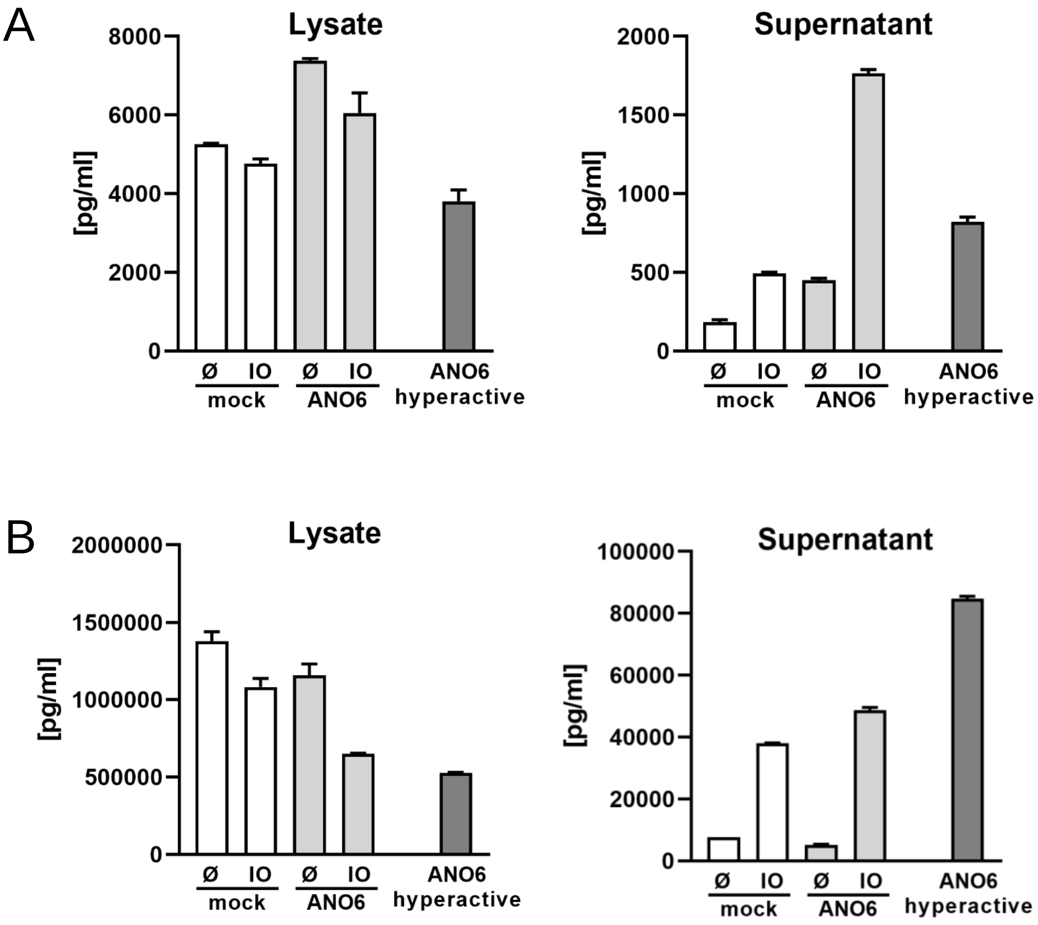

Supplementary Figure S4: Anoctamin-6 (ANO6) modulates CD137 shedding. One representative experiment out of five **(A)** and three **(B)** ELISAs (shown normalized in Figure 4A/B) is depicted to show the absolute levels of CD137 in cell lysates and supernatants of HT29 **(A)** and HEK cells **(B)**, respectively (mean +/- SD).
ANO6 and mock-transfected HT29 cells or HEK293T WT cells were stimulated with ionomycin (IO; 1 µM) for 30 min. In addition, cells co-transfected with CD137 and hyperactive ANO6 were analyzed in the absence of any stimulus for 30 min by CD137 ELISA.

Supplementary Figure S5

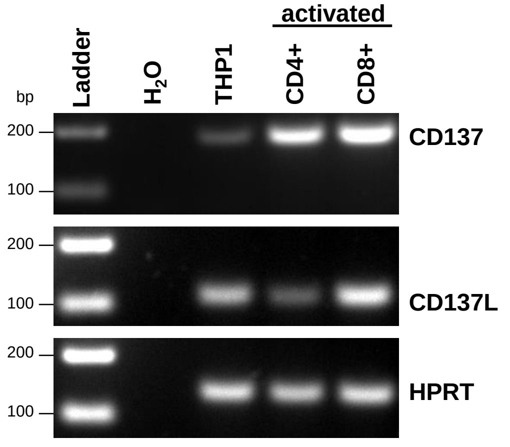

Supplementary Figure S5:
Representative agarose gel images showing mRNA expression of CD137 and CD137 Ligand (CD137L) in activated CD4+/CD8+ T cells. Total RNA was extracted and subjected to cDNA synthesis. The PCR products were amplified using specific primers and analyzed using 2,5 % agarose gel electrophoresis. THP1 cells were used as positive control. The housekeeping gene HPRT served as internal control.
